# Genome of the endangered Guatemalan Beaded Lizard, *Heloderma charlesbogerti*, reveals evolutionary relationships of squamates and declines in effective population sizes

**DOI:** 10.1093/g3journal/jkac276

**Published:** 2022-10-13

**Authors:** Carl J Dyson, Aaron Pfennig, Daniel Ariano-Sánchez, Joseph Lachance, Joseph R Mendelson III, Michael A D Goodisman

**Affiliations:** School of Biological Sciences, Georgia Institute of Technology, Atlanta, GA 30332, USA; School of Biological Sciences, Georgia Institute of Technology, Atlanta, GA 30332, USA; Centro de Estudios Ambientales y Biodiversidad, Universidad del Valle de Guatemala, Zona 15 01015, Guatemala; Heloderma Natural Reserve, Zacapa 19007, Guatemala; School of Biological Sciences, Georgia Institute of Technology, Atlanta, GA 30332, USA; School of Biological Sciences, Georgia Institute of Technology, Atlanta, GA 30332, USA; Zoo Atlanta, Atlanta, GA 30315, USA; School of Biological Sciences, Georgia Institute of Technology, Atlanta, GA 30332, USA

**Keywords:** Helodermatidae, Gila Monster, comparative genomics, phylogenomics, effective population size, endangered species

## Abstract

Many lizard species face extinction due to worldwide climate change. The Guatemalan Beaded Lizard, *Heloderma charlesbogerti*, is a member of the Family Helodermatidae that may be particularly imperiled; fewer than 600 mature individuals are believed to persist in the wild. In addition, *H. charlesbogerti* lizards are phenotypically remarkable. They are large in size, charismatically patterned, and possess a venomous bite. Here, we report the draft genome of the Guatemalan Beaded Lizard using DNA from a wild-caught individual. The assembled genome totals 2.31 Gb in length, similar in size to the genomes of related species. Single-copy orthologs were used to produce a novel molecular phylogeny, revealing that the Guatemalan Beaded Lizard falls into a clade with the Asian Glass Lizard (Anguidae) and in close association with the Komodo Dragon (Varanidae) and the Chinese Crocodile Lizard (Shinisauridae). In addition, we identified 31,411 protein-coding genes within the genome. Of the genes identified, we found 504 that evolved with a differential constraint on the branch leading to the Guatemalan Beaded Lizard. Lastly, we identified a decline in the effective population size of the Guatemalan Beaded Lizard approximately 400,000 years ago, followed by a stabilization before starting to dwindle again 60,000 years ago. The results presented here provide important information regarding a highly endangered, venomous reptile that can be used in future conservation, functional genetic, and phylogenetic analyses.

## Introduction

Lizards face significant challenges to their survival due to ongoing climate change (Sinervo *et al.* 2010). Many lizard species are already imperiled. For example, the charismatic lizard Family Helodermatidae contains a single genus *Heloderma*, which comprises 5 extant species. Of these species, the Guatemalan Beaded Lizard, *Heloderma charlesbogerti*, has the smallest range and suffers from multiple anthropogenic threats (Beck 2005; Ariano-Sánchez and Gil-Escobedo 2021).

*Heloderma* lizards are distinctive by their rather large size, heavily ossified scales, and often bright coloration (Beck 2005). Helodermatid lizards fossils are known from at least ∼35 MYA, with strong morphological conservatism over time (Reiserer *et al.* 2013), making them prime examples of living fossils. Perhaps the most well-known *Heloderma* species is the Gila Monster (*Heloderma suspectum*), from the southwestern United States and adjacent Mexico. The 4 other *Heloderma* species generally are similar in appearance and occur in Mexico and Guatemala. Helodermatid lizards are dangerously and famously venomous. However, human envenomations are rare and not fatal, as *Heloderma* venom is believed to serve a defensive purpose instead of a predatory one (Beck 2005; Ariano-Sánchez 2008).

**Fig. 1.**
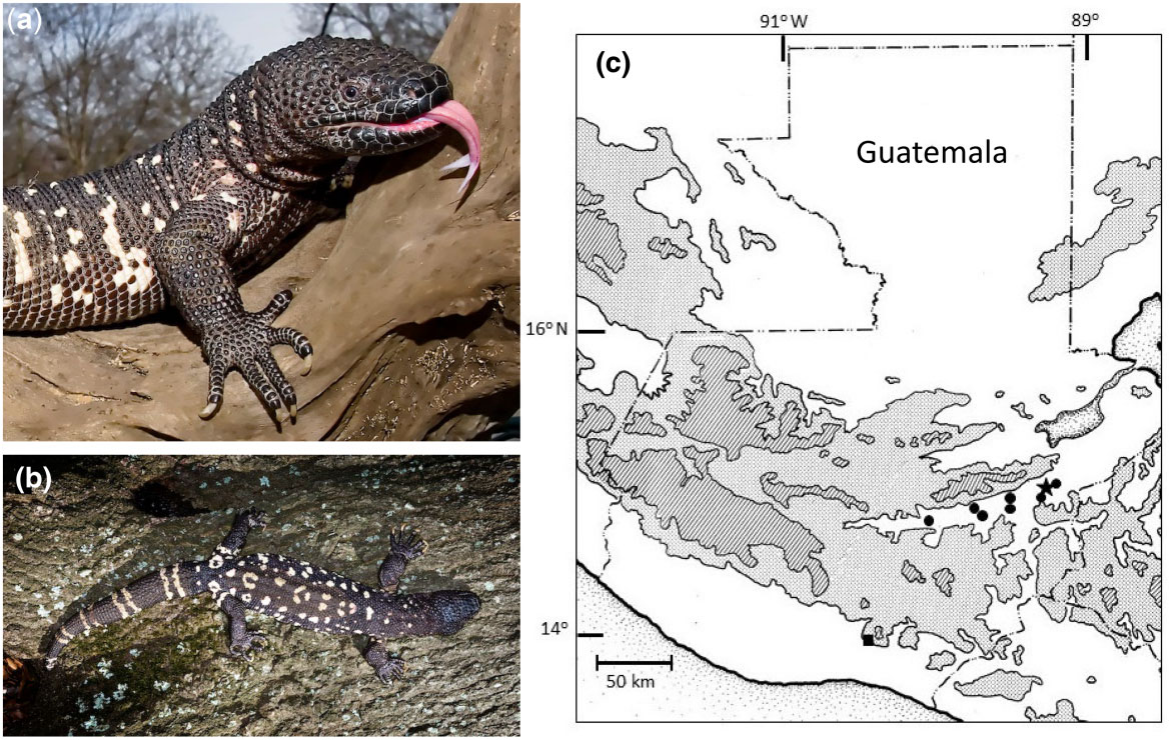
a, b) The Guatemalan Beaded Lizard, *Heloderma charlesbogerti*, is a highly endangered species from the venomous lizard family Helodermatidae. Photocredit: M. D. Kern. c) It is currently found only in the isolated xeric habitat of the Motagua Valley on the Atlantic versant of eastern Guatemala. Circles indicate documented localities for the Guatemalan Beaded Lizard within the Motagua valley. The star denotes the collection site of the individual whose genome was sequenced in this study. The square demarks the collection from a relic population on the Pacific versant.

The Guatemalan Beaded Lizard is the most recently described helodermatid lizard (Campbell and Vannini 1988; Reiserer *et al.* 2013) (Fig. 1, a and b). It is currently found only in the isolated xeric habitat of the Motagua Valley in the Atlantic Versant of eastern Guatemala (Fig. 1c; CONAP 2020; Ariano-Sánchez and Gil-Escobedo 2021). The Motagua Valley is a narrow strip of xeric habitat bounded to the west and south by upland pine-oak forest and to the north by the very steep Sierra de las Minas that supports cloud forest on its main ridge. To the east, as the Sierra de las Minas decreases in elevation, the xeric vegetation of the Motagua Valley grades into lowland rainforest that does not support populations of *Heloderma*. Consequently, the entire natural range of this species is limited to only about 20,000 hectares (CONAP 2020). Moreover, ongoing habitat alterations from agriculture and urbanization have greatly decreased the already small amount of habitat available to this species, with contemporary populations limited to a few isolated sites that are formally or informally protected (Ariano-Sánchez 2006; Domiguez-Vega *et al.* 2012).

As a result of its naturally small distribution, severe local habitat alteration, and persecutive killing by humans, the Guatemalan Beaded Lizard is considered to be one of the most endangered lizard species. Only 500–600 mature individuals are believed to exist in the wild (CONAP 2020). Alongside these threats, global warming is expected to negatively impact this species by reducing the width of their operational thermal window (Ariano-Sánchez *et al.* 2020, 2022).

In this study, we present a draft genome of the Guatemalan Beaded Lizard. The genome will provide important information on the evolutionary history of the Helodermatidae and related lizard species. In addition, our work in developing a draft genome of this critically endangered species can contribute to conservation efforts by providing information on historical changes in population size, and help further our understanding of an understudied taxon within the venomous lizards (Paez *et al.* 2022).

## Methods

### Sample acquisition

Zoo Atlanta in Atlanta, Georgia, USA, maintains a conservation-breeding colony of Guatemalan Beaded Lizards comprising approximately 40 individuals; the colony includes several founder individuals that were wild-caught in the 1980s. The focal Guatemalan Beaded Lizard whose genome was sequenced was a wild-caught individual that is a paratype of the type series (Campbell and Vannini 1988). The individual was an adult male collected at adult size in 1984 in the vicinity of the village of El Bejucal, ca. 12-km SSE of the Gualan, Departamento Zacapa, Guatemala. It was maintained at Zoo Atlanta from 2000 until its death from natural causes in 2020. The voucher specimen is UTA R-15002 and is maintained at the Amphibian and Reptile Diversity Research Center at the University of Texas at Arlington. Sampling was approved by the Zoo Atlanta Scientific Research Committee.

### Sequencing

Blood was previously drawn from the focal Guatemalan Beaded Lizard male specimen by Zoo Atlanta personnel during an unrelated medical procedure. The blood sample was treated with potassium and EDTA for preservation and frozen at –20°C to prevent degradation. DNA was extracted from the blood sample using an Omega Biotek E.Z.N.A. Tissue DNA Kit (catalog # D3396). Standard kit procedures were followed except that the cell lysis stage was undertaken with lower volumes of blood to avoid issues with blood clotting interfering with column purification. Extracted DNA was tested for purity and concentration using a NanoDrop spectrophotometer. DNA concentrations of genomic DNA samples were estimated to be ∼40–50 ng/μl. A total of ∼7 μg of genomic DNA was ultimately used for DNA sequencing.

Genomic DNA was sorted for high-quality, long sequences using BluePippin size selection before preparation for PacBio sequencing. Two sequencing insert libraries were created: a traditional long-read library consisting of >30-kb sequences, and a library of 15- to 20-kb “HiFi Read” sequences, which yield shorter, more accurate reads (>99% accuracy). Libraries then underwent on-site quality assessment and cleanup prior to sequencing. Finally, the libraries were sequenced on 2 runs of PacBio Sequel II SMRTCell sequencing.

#### Genome Assembly

Prior to assembly, the bioinformatics tool Jellyfish v2.3.0 (Marcais and Kingsford 2011) was used to provide estimates of k-mer frequency and sequencing read distribution of the PacBio long reads (Institute for Systems Genomics 2022). This was performed with a minimum threshold quality value of 20 in order to exclude possible sequencing errors. Jellyfish was then run across a range of potential k-mer values ranging from 14 to 31 to more efficiently capture the optimal value within the reads. Resulting k-mer distributions were used to produce approximations of genome size in R using the equation *k_c_* = (*L* − *k*) + 1, where *L* is the genome size approximation and *k_c_* is the count of k-mers of size *k*.

PacBio raw long reads were assembled using the Flye v2.8.1-b1676 de novo assembler (Kolmogorov *et al.* 2019). The de novo assembly was run using the default program parameters, aside from manual input of the genome size approximation (2.25 Gb, from Jellyfish estimation) and a minimum overlap length of 10 kb. The initial genome assembly was then run through 3 iterations of polishing using the in-suite polisher to optimize read alignments.

Following genome assembly, quality assessment was performed using QUAST v5.1.0rcl (Gurevich *et al.* 2013) (Supplementary Fig. 1). The analysis was run using the *large* parameter flag, which optimizes several parameters such as the sequence source (eukaryotic), the minimum contig length threshold (3,000 bp), the minimum alignment threshold (500 bp), and the extensive misassembly size for relocations (7,000 bp). Genome contiguity statistics such as number of contigs, contig N50, and contig L50 were obtained to better illustrate the quality of the draft genome.

The completeness of the draft genome for the Guatemalan Beaded Lizard was assessed by Benchmarking Universal Single-Copy Orthologs (BUSCOs, v5.2.2) (Simao *et al.* 2015). The database vertebrata_odb10.2019-11-20 consisting of 3,354 orthologous genes was used to identify putative single-copy orthologs within the assembly. BUSCO results were compared to other related lizard taxa to assess the quality of the new genome assembly.

#### Genome Annotation

Prior to genome annotation, repeats were de novo softmasked using RepeatModeler (Flynn *et al.* 2020), RepeatMasker (Smit *et al.* 2022), and Tandem Repeat Finder (TRF) (Benson 1999). A custom repeat library was built for the draft genome of the Guatemalan Beaded Lizard with RepeatModeler v2.0.3 using the rmblast v2.11.0 search engine (Altschul *et al.* 1990). The custom repeat library and the Dfam database were then used to annotate repeats with RepeatMasker v4.1.2 (Storer *et al.* 2021; Smit *et al.* 2022). Because RepeatMasker does not annotate tandem repeats with a period greater than ten, TRF v4.09.1 was used to identify tandem repeats with a period of up to 500. The repeat annotation of RepeatMasker and TRF were then combined, merging overlapping intervals. Based on this repeat annotation, the draft genome was softmasked.

We chose BRAKER2 v2.1.6 for the prediction of protein-coding genes because it was designed for cases when RNA-Seq data and annotation of very closely related species are absent. In addition to the soft-masked draft genome, we supplied BRAKER2 with OrthoDB v10, from which it automatically generated extrinsic protein hints for the training of GeneMark-EP and AUGUSTUS (Lomsadze *et al.* 2005; Stanke *et al.* 2006; Kriventseva *et al.* 2019; Bruna *et al.* 2020, 2021). The completeness of the predicted proteome was assessed with BUSCO (Simao *et al.* 2015), and predicted protein sequences were functionally annotated using InterProScan version 55.5-88.0. In addition, tRNAscan-SE v2.0.9 was used to identify tRNA genes within the draft genome (Chan *et al.* 2021). Predicted tRNA genes that overlapped by more than 50% with an annotated repeat region were removed. A Snakemake workflow implementing the genome annotation can be found at https://github.com/LachanceLab/GBL (Mölder *et al.* 2021).

### Phylogenetic analysis

OrthoFinder v2.3.8 was used to find orthologous groups between 13 reptile species, including the new draft genome of the Guatemalan Beaded Lizard (Emms and Kelly 2019). In the event that multiple reference genomes were available for a species, we selected 1 assembly for use in analysis based on quality and recency of publication (Supplementary Table 1). OrthoFinder was run with optional parameters *-M msa -A muscle -T iqtree*, where msa represents the method for gene tree inference, MUSCLE v5.1 is the program used for the multiple sequence alignment (MSA), and IQ-TREE v2.2.0 is used for tree inference from those alignments.

Phylogenetic construction was performed to better understand the position of the Guatemalan Beaded Lizard among 12 other related reptile species (Alföldi *et al.* 2011; Castoe *et al.* 2013; Wang *et al.* 2013; Green *et al.* 2014; Badenhorst *et al.* 2015; Georges *et al.* 2015; Liu *et al.* 2015; Song *et al.* 2015; Xiong *et al.* 2016; Gao *et al.* 2017; Lind *et al.* 2019) (Supplementary Table 1). Because of the significant length of evolutionary history that this tree encompassed, the genes chosen for use in building the tree were made up of 3,324 BUSCO single-copy orthologs from the Vertebrata dataset that were found to be present in at least 3 of the 13 species. Sequences of the orthologous genes were aligned using default parameters within PRANK v170427 (Loytynoja 2014) and combined into a super-matrix for further gene tree analysis. Maximum likelihood phylogenies were created using MEGA-X v10.2.6 using the JTT matrix-based model and were run with 500 bootstrap replications.

### Molecular evolution of genes

To test whether specific genes evolved at a different rate in the Guatemalan Beaded Lizard, we compared the ratio of nonsynonymous to synonymous nucleotide substitution (*dN*/*dS*) on the branch leading to the Guatemalan Beaded Lizard with that leading to the Asian Glass Lizard, *Dopasia gracilis*, and the Komodo Dragon, *Varanus komodoensis*, using the Green Anole, *Anolis carolinensis*, as an outgroup.

First, the genomic assembly and corresponding annotations in GFF format were obtained for the related species (Supplementary Table 1). Nucleotide sequences of complete coding regions (i.e. both start codon and terminal stop codon present) were extracted and translated with gffread v0.12.7 (Pertea and Pertea 2020). Orthologs were then identified on a protein level using OrthoFinder v2.5.4 (Emms and Kelly 2019). For the subsequent analysis, only single-copy orthologous sequences were considered. For those single-copy orthologs, MSA was performed on protein-level using Clustal Omega v1.2.4 (Sievers *et al.* 2011). The obtained protein alignments were then reverse translated to obtain codon-specific alignments of nucleotide sequences. Reverse translating protein alignments is possible because for each amino acid, the corresponding codon in the nucleotide sequence is known from the original nucleotide and amino acid sequences. The codon-specific alignments were then concatenated, and a phylogenetic tree of the above 4 species was built with IQ-TREE v2.2.0-beta (Minh *et al.* 2020). *dN*/*dS* ratios were then estimated from the codon-specific alignments for each gene using the *codeml* program in the PAML package version 4.9 (Yang 2007).

To determine whether a gene evolved at a differential rate in the phylogenetic tree, 2 models were fitted: (1) a simple model that does not allow selection and calculates 1 *dN*/*dS* ratio for the entire tree (*model = 0*) and (2) a more complex model that calculates a *dN*/*dS* ratio for each branch in the phylogenetic tree (*model = 1*). A likelihood ratio test (LRT) was performed to determine whether the more complex model provided a significantly better fit than the simple model. The more complex model was accepted if the LRT statistic, that is, 2*x* (*l*_1_ − *l*_0_), was greater than the critical X^2^ test statistic for *k = *1 degrees of freedom on a Bonferroni corrected significance level of 0.01 (*ɑ* = 0.01/*N*, where *N* is the number of tested single-copy orthologs).

Gene names of single-copy orthologs from the Green Anole were extracted and used for pathway analysis, because the genome of the Green Anole is well annotated. We used DAVID (2021 Update) (Jiao *et al.* 2012) to perform a pathway analysis of genes that evolved at different rates in the phylogenetic tree and whose *dN*/*dS* ratio was in the bottom or top 10% among *dN*/*dS* ratios specific to the branch leading to the Guatemalan Beaded Lizard. The KEGG database was used for the pathway analysis (Kanehisa *et al.* 2016), and the list of all single-copy orthologs was used as background. Snakemake workflows for these analyses can be found at https://github.com/LachanceLab/GBL (Mölder *et al.* 2021).

### Venom genes

One of the most well-known traits of helodermatid lizards is the production of venom used in defensive responses. The venom composition previously was found to be conserved among 3 of the 5 helodermatid species and also reported to have similarities to viper venoms (Koludarov *et al.* 2014; Mackessy 2022). For these reasons, we built a custom database of venom proteins, comprising 20 known venom peptides/proteins from *Heloderma* species reported in Table 1 of Koludarov *et al.* (2014) and 1232 viper venoms that were retrieved from the UniProtKB/Swiss-Prot database through https://venomzone.expasy.org/ (UniProt Consortium 2021). Using jackhmmer v3.1b2, we searched for putative venomous proteins among the annotated protein sequences based on similarity to venom proteins included in our custom database (Mistry *et al.* 2013). We used Bonferroni corrections so that only hits with e-values smaller than 0.0001/*n* (where *n* is the number of queries) for the full query and e-values smaller than 0.01/*n* for the single best-scoring domain envelope were retained.

**Table 1.** Summary statistics of the assembled Guatemalan Beaded Lizard draft genome.

Metric	Genome
Genome size (bp)	2,308,465,658
Coverage	86×
Number of contigs	3,551
Contigs ≥50 kb	2,801
Longest contig (bp)	7,420,054
Contig N50	1,358,783
Contig N90	389,083
Contig L50	517
Contig L90	1,704
GC (%)	45.05

Proteins of known toxin classes in helodermatid lizards that were missing after the initial search with jackhmmer were aligned to the draft genome using Exonerate v2.4.0 in *protein2genome* mode, requiring at least 50% protein match (Slater and Birney 2005). Computer code for these analyses can be found at https://github.com/LachanceLab/GBL.

### Effective population size

The effective population size (*N_e_*) over time of the Guatemalan Beaded Lizard was inferred using Pairwise Sequential Markovian Coalescent (PSMC) v0.6.5 (Li and Durbin 2011). In brief, the raw long reads were aligned to the assembled genome using pbmm2 v1.30.0. To reduce the impact of sequencing error, homopolymers (i.e. low complexity regions of repetitive nucleotides) were collapsed using the *–collapse homopolymers* flag. Variant calling was conducted with longshot v0.4.1 (Edge and Bansal 2019), a variant caller specifically designed for (noisy) long-read data. To further reduce the impact of low-quality calls on heterozygous sites, strict variant filtering was applied using bcftools v1.14-36 (Danecek *et al.* 2021). Only variants with a Phred scaled quality score (QUAL) ≥60 and raw read depth (DP) ≥40 were retained for subsequent analysis. Furthermore, variants for which less than 99% of the reads aligned with a mapping quality of at least 20 (MQ20) were removed. After the variant filtering, the fraction of heterozygous sites, i.e. heterozygosity, was 1.454 × 10^−4^.

ROH filtering was applied to account for possible recent inbreeding in the wild population from which this individual was sampled, as recent inbreeding can affect PSMC estimates. ROHs were detected using a hidden Markov model implemented in bcftools (Narasimhan *et al.* 2016), which requires a default allele frequency to be specified when a single individual is analyzed. We tried 0.1 and 0.4 as allele frequency thresholds and found that these thresholds did not affect the ultimate outcome of the PSMC. As 0.1 is the more conservative threshold, masking more sequence, it was chosen as the default allele frequency. Variants falling into ROHs were removed from the set of called variants, and ROHs were hardmasked in the homopolymer collapsed sequence using bedtools v2.30.0 (Quinlan and Hall 2010). This marginally increased the heterozygosity from 1.454 × 10^−4^ to 1.465 × 10^−4^.

A consensus sequence was then constructed, considering only contigs with a length of at least 500 kb and minimum heterozygosity of 10^−5^ using bcftools (Danecek *et al.* 2021). The selected contigs had a slightly lower mean heterozygosity of 1.438 × 10^−4^. Based on this consensus sequence, the input for the PSMC (Li and Durbin 2011) was then prepared using the *fq2psmcfa* script, and 100 bootstrap replicates were generated using the *splitfa* script. Generally, the PSMC results were robust to various parameterizations of the PSMC. To avoid under- and overfitting, at most 25 iterations (*-N*) were run. Because the Guatemalan Beaded Lizard has lower heterozygosity than humans, a lower maximum 2N_0_ coalescent time of 10 (*-t*) was chosen. For the same reason, shorter time intervals and more free interval parameters were chosen than recommended for humans (*-p* “2 + 40*2 + 4 + 6”). The default parameterization of the initial ratio of the scaled mutation and recombination rate was used (*-r5*).

Finally, time and *N_e_* were scaled using a generation time of 12.3 years and mutation rate (µ) of 9.47 × 10^−9^ per site per generation (12.3 years per generation × 7.7 × 10^−10^ per site per year) (Perry *et al.* 2018). We estimated the generation to be 12.3 years using the IUCN Red List method 3. According to this method, the generation time is the age of first reproduction plus a constant *z* times the length of the reproductive period (IUCN Standards and Petitions Committee 2022). The constant *z* is generally estimated to be approximately 0.3 for vertebrates (Pacifici *et al.* 2013; Keith *et al.* 2015). For the Guatemalan Beaded Lizard, the age of first reproduction is approximately 3 years, and the longevity is approximately 34 years in the wild, with individuals being fertile their entire life. Thus, the generation time of the Guatemalan Beaded Lizard is: 3 years + 0.3 × (34 years − 3 years) = 12.3 years. A snakemake workflow of the analysis is available at https://github.com/LachanceLab/GBL (Mölder *et al.* 2021). Using the observed heterozygosity (*H*) as a proxy for the population mutation rate (*θ* = 4 *N_e_*µ), we approximated the current value of *N_e_*. The relationship between *H* and *θ* is given by *H* = *θ*/(*θ* + 1).

## Results and discussion

### Genome assembly

We assembled and analyzed the genome of the highly endangered Guatemalan Beaded Lizard. The initial PacBio long-read sequencing resulted in 232 Gb of raw reads, with a total coverage of 86× across the draft assembly (Table 1). A number of these reads were PacBio HiFi reads, which have higher read accuracy (> 99%) than traditional long-read sequencing technology. The assembled genome was 2.31 Gb in length. The assembly comprised 3,551 contigs, 83% of which were 50 kb or larger in size. Evaluation of the draft assembly produced a contig N50 of 1,358,783 bp. The overall GC content of the genome was estimated to be 45.05%.

The genome size of the Guatemalan Beaded Lizard is somewhat larger than the sizes of other closely related lizard species (Table 2). However, the divergence time of the Guatemalan Beaded Lizard from these other taxa is well over 100 MYA (Simões and Pyron 2021) and thus some variation in genome size is expected. Regardless, the genome size of the Guatemalan Beaded Lizard falls within the upper range of the overall clade of squamates which generally spans from 1.5 to 2.5 Gb (Table 2).

**Table 2.** Basic genome statistics in the Guatemalan Beaded Lizard, *H. charlesbogerti*, and related squamate reptiles.

Species	Contigs	Contig >50 kb	Coverage ×	Length (Gb)	GC %	Contig N50	Contig N90	Contig L50	Contig L90
*H. charlesbogerti*	3,551	2,801	86	2.31	45.03	1,358,783	389,083	517	1,704
*V. komodoensis*	1,411	423	144	1.51	44.04	23,831,982	3,205,276	17	80
*D. gracilis*	3,811	2,539	154	1.78	43.71	1,280,496	299,089	407	1,481
*S. crocodilurius*	5,434	2,584	70	2.00	44.35	1,791,797	280,501	310	1,372
*A. carolinensis*	6,457	1,187	7	1.80	40.32	1.51E + 08	408,349	5	416

**Fig. 2.**
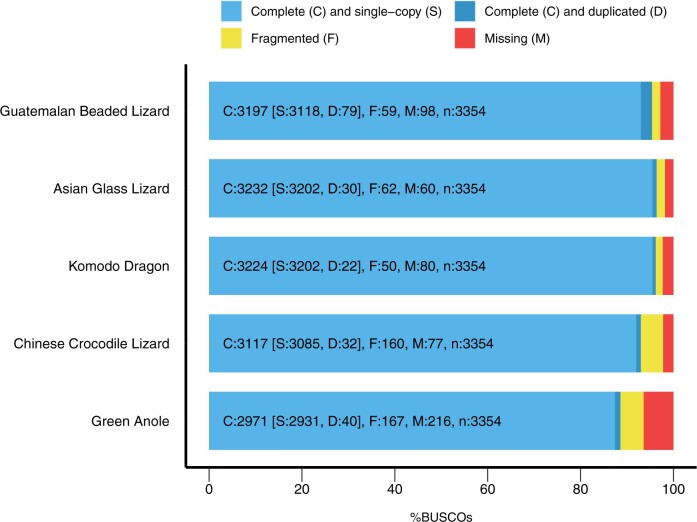
Comparison of BUSCO genome completeness measures between the Guatemalan Beaded Lizard and 4 other related lizard species. Results were obtained using a set of 3,354 orthologous genes from the BUSCO dataset Vertebrata_odb10.

Our genome assembly for the Guatemalan Beaded Lizard appeared to be robust based on the presence of Benchmarking Universal Single-Copy Orthologs (BUSCOs) (Simao *et al.* 2015). We found 93.0% of complete, single-copy BUSCO orthologs from the Vertebrata dataset, as well as 2.4% and 1.8% of BUSCOs that were duplicated or fragmented, respectively (Fig. 2). Our BUSCO results were similar to the genome assemblies of other closely related species (Fig. 2). This underscores the power of long-read sequencing technology for de novo genome assembling, which was recently impressively demonstrated with a telomere-to-telomere assembly of the human genome (Nurk *et al.* 2022).

### Genome content

Approximately 57.54% of the genome consisted of identifiable repetitive DNA, which is consistent with the repeat content previously reported for other squamates (Pasquesi *et al.* 2018). Retrotransposons were more common than DNA transposons (23.83% vs 2.59%), with long interspersed nuclear elements (LINEs) being the most abundant class. LINEs comprised 20.81% of the genome, while short interspersed nuclear elements and long terminal repeats occupied only 1.41% and 1.61% of the genome, respectively (Supplementary Table 2).

We performed ab initio gene prediction supported by extrinsic protein evidence from OrthoDBv10. Our analyses resulted in 31,411 protein-coding genes and 32,205 distinct mRNAs being identified within the genome. The average gene had 4 exons with an average exon length of 231 bp and an average intron length of 1,967 bp. The total number of identified proteins (32,205) in the Guatemalan Beaded Lizard genome was comparable to the number identified in the Green Anole genome (34,814) (Alföldi *et al.* 2011) but was higher than the number identified in the Asian Glass Lizard genome (19,513) (Song *et al.* 2015) and lower than the number identified in the genome of the Komodo Dragon (39,545) (Lind *et al.* 2019) (the number of proteins for each species were obtained from corresponding annotation files; see Supplementary Table 1 for accession IDs). We found that 89.81% (28,923/32,205) of the predicted proteins have at least 1 InterPro annotated functional domain (Jones *et al.* 2014; Blum *et al.* 2021). In addition, our analyses indicated the presence of 337 tRNA genes and 208 tRNA pseudogenes after filtering out predicted tRNA genes that significantly overlapped with previously annotated repeat regions.

#### Phylogeny

The Family Helodermatidae contains the single genus *Heloderma* (Bogert and Martín del Campo 1956). The placement of the Helodermatidae among the other squamate reptiles is of substantial interest for understanding the evolution of the beaded lizards and other squamates (Simões and Pyron 2021). Thus, we investigated the phylogenetic relationship between the Guatemalan Beaded Lizard and other snakes and lizards in order to better understand the evolutionary history of this clade.

**Fig. 3.**
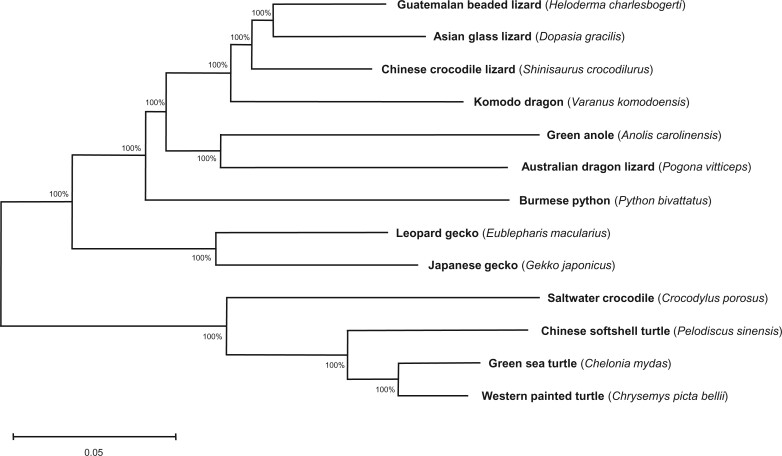
Maximum likelihood species tree representing first phylogenetic placement of the Guatemalan Beaded Lizard from genomic data. Alignments were performed using 3,354 BUSCO orthologs to determine evolutionary divergence. Inset bootstrapping values represent percentage of trees in which clustered taxa were found across 500 replications. Branch lengths are to scale, with the scale bar representing 0.05 nuclear substitutions per site.

A phylogeny created using the draft genome of the Guatemalan Beaded Lizard and 12 other related reptile species indicated that the helodermatids fall into a clade with the Asian Glass Lizard (Anguidae), and in close association with the Komodo Dragon (Varanidae) and the Chinese Crocodile Lizard (Shinisauridae) (Fig. 3). This represents the approximate expected position of the Helodermatidae based on prior related phylogenetic analyses (Lind *et al.* 2019).

### Molecular evolution

We investigated the evolutionary constraint (*dN*/*dS*) of genes within the Guatemalan Beaded Lizard genome in order to better understand ongoing evolutionary processes. The *dN*/*dS* ratio was calculated from MSAs of 1,832 single-copy orthologs of the Guatemalan Beaded Lizard, the Asian Glass Lizard, the Komodo Dragon, and the Green Anole. A gene was considered to have evolved at a different rate in the phylogenetic tree if a branch model had a significantly higher likelihood than a simple model (see *Methods*). Our results indicated that 504 genes had a different amount of evolutionary constraint on the branch leading to the Guatemalan Beaded Lizard (Bonferroni corrected *P*-value <5.46 × 10^−6^) (Supplementary Table 3). Of those, genes that exhibited lower constraints on the branch leading to the Guatemalan Beaded Lizard (i.e. their *dN*/*dS* ratio was in the top 10% of *dN*/*dS* ratios specific to the branch leading to the Guatemalan Beaded Lizard) were enriched for proteins involved in the KEGG pathway N-glycan biosynthesis (*P = *0.013; FDR = 0.45) and metabolic pathways (*P = *0.037; FDR = 0.62), although their significance did not withstand FDR correction. No pathway enrichment was found for genes with higher evolutionary constraints (i.e. their *dN*/*dS* ratio was in the bottom 10%).

### Venom genes

Many members of the squamates use venom in predation or defense (Vonk *et al.* 2013). Helodermatids are believed to use venom mainly for defense (Beck 2005). We identified 312 protein-coding regions that had significant similarities to known venom proteins in helodermatid lizards and vipers. Previously, Koludarov *et al.* (2014) found that the venom composition is conserved among helodermatid lizards, and consists of 6 main protein types/toxin classes [i.e. cysteine-rich secretory protein (CRiSP), Exendin, Helofensin, Kallikrein, B-type Natriuretic peptide/helokinestatin precursor, and Phospholipase A_2_ Type III; see Table 1 in Koludarov *et al.* (2014)]. The genome of the Guatemalan Beaded Lizard contained genes with significant similarities to associated proteins in 5 of the 6 known helodermatid toxin classes, lacking only proteins similar to members of the Helofensin Class. To check whether a gene encoding a Helofensin-like protein was indeed missing in the Guatemalan Bead Lizard genome, we reverse aligned protein instances of this class to the draft assembly. We found 1 instance of a Helofensin-type protein likely to be present in the draft genome, which was likely missed by the gene prediction algorithm due to its short exons. Notably, we also found 3 genes that exhibited significant similarities to viper Reticulocalbin-2, Zinc metalloproteinase/disintegrin and Cystatin, respectively, and showed evidence of weak positive selection (i.e. *dN*/*dS* ratios of 999, 1.18, and 1.08). While the role of Cystatin in venoms is unknown, Reticulocalbin—a calcium binding protein—is hypothesized to interact with Phospholipase A2 (Dodds *et al.* 1995), and Zinc metalloproteinase/disintegrin inhibits platetet aggregation. We point out that Exendin-4, a protein causing hypotension, also plays a key role in the metabolic control of glucose levels (Young *et al.* 1999) and has been synthesized based on saliva from the Gila Monster (*H. suspectum*) for use in humans with diabetes (Nielsen *et al.* 2004). However, all other genes with similarities to known venom proteins and whose *dN*/*dS* was estimated during the above analysis had inferred *dN*/*dS* ratios of less than 0.75 on the branch leading to the Guatemalan Beaded Lizard. This is in line with the observed conservation of venoms in helodermatid lizards (Koludarov *et al.* 2014; Mackessy 2022).

### Estimates of historical effective population size

**Fig. 4.**
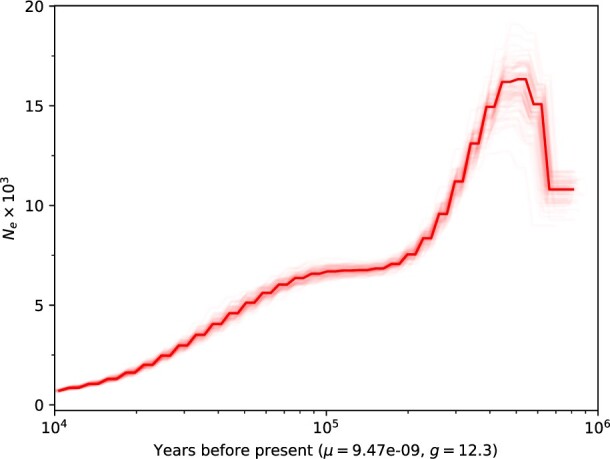
PSMC estimates of the historical effective population size (*N_e_*) in the Guatemalan Beaded Lizard with bootstrap intervals (lighter lines) under the assumption of a generation time of 12.3 years and a mutation rate of 9.47 × 10^−9^ per site per generation. Note that the scale on the *y*-axis (the estimated *N_e_*) is dependent on the estimated mutation rate and generation time, but the trajectory of *N_e_*, and the estimated timings of the declines, is independent of these variables.

The Guatemalan Beaded Lizard is one of the most endangered lizards in the world (Ariano-Sánchez 2006). Results from PMSC temporal estimations of effective population size (*N_e_*) in this species display a decline approximately 400,000 years BP (Before the Present), followed by a stabilization around 200,000 years BP before dwindling again 60,000 years BP to a small *N_e_* 10,000 BP (Fig. 4). We note that the PMSC estimates of *N_e_* depend on the assumed mutation rate (9.47 × 10^−9^ per site per generation), which in turn is contingent on the estimated generation time (*g* = 12.3 years). Lower generation times lead to a lower mutation rate, and hence to a greater value of *N_e_*. However, because the time scaling depends on the ratio of generation time and per generation mutation rate, which is constant in our analyses due to the interdependency of the generation time and mutation rate, changing the generation time and rescaling the mutation rate accordingly does not affect the timings of declines in *N_e_* inferred by the PMSC.

The small *N_e_* estimate of the Guatemalan Beaded Lizard is consistent with the low heterozygosity of 1.454 × 10^−4^. For comparison, the heterozygosity of the Komodo Dragon, another critically endangered lizard, is on the same order of magnitude, ranging from 7.62 × 10^−5^ to 1.31 × 10^−4^ (Iannucci *et al.* 2021). Based on the observed heterozygosity and population-genetics theory (Kimura and Crow 1964), the *N_e_* of the Guatemalan Beaded Lizard is estimated to be approximately 3,839. Note that this estimate reflects historically larger population sizes and does not equate to the actual population size in the wild, which is estimated by conservationists and ecologists to be approximately 600 (CONAP 2020).

The estimates of declines in *N_e_* of the Guatemalan Beaded Lizard are consistent with known patterns of biogeography. Three records of this species were reported from xeric habitat on the Pacific Versant of Guatemala: 2 near Lago de Amatitlan and 1 in the southeastern portion of the Pacific lowlands (Anzueto and Campbell 2010). However, these populations are now presumed to be extinct (Ariano-Sánchez and Gil-Escobedo 2021). Regardless, these records support a Pacific-corridor hypothesis, which suggests that *Heloderma* reached Motagua Valley by dispersing along continuous dry forest habitat from the Pacific coast of Mexico through the Pacific coast of Guatemala, thus entering Motagua Valley at the xeric habitat surrounding Lago de Amatitlan (Ariano-Sánchez and Salazar 2007; Anzueto and Campbell 2010). This area, known as the Amatitlan cauldron, had intense volcanic activity between 300,000 and 23,000 BP (Wunderman and Rose 1984). Ancient populations of *Heloderma* probably reached Motagua Valley through the dry forest surrounding the Amatitlan cauldron, based on the evidence of a dispersal route from the Pacific coast (Anzueto and Campbell 2010). We suggest that the massive eruptions may have contributed to the sharp past decline in *N_e_* of the Guatemalan Beaded Lizard identified in our analyses. A recent dispersal to Motagua Valley, preceded by a decline in *N_e_*, likely contributed historically to the rarity of the species.

Another observation that supports the hypothesis of a recent dispersal to Motagua Valley comes from archaeological evidence from ancient human populations. The absence of any pictorial representations of the Guatemalan Beaded Lizard in Mayan ceramics from Motagua Valley is surprising, taking into account that Mayans occupied Motagua Valley around 2,500 years BP for at least 2,000 years and are well known for documenting their surrounding biodiversity (Romero 2007). In comparison, there are several pictorial representations of related species of *Heloderma* from Mexico and the United States in many pre-Columbian societies (Del Campo and Sanchez 1936; Beck 2005). Consequently, the Guatemalan Beaded Lizard may have only been present in its contemporary habitat very recently in geological time. This highlights the possibility that the Guatemalan Beaded Lizard had always been a rare species at Motagua Valley, becoming much rarer in the last century likely due to habitat destruction, persecutive killing, and smuggling.

### Conclusions

We present here the draft genome of the Guatemalan Beaded Lizard, *H. charlesbogerti*. Our results provide molecular evidence for the placement of this endangered species within the squamate phylogeny. Additionally, we provide information regarding the evolutionary divergence of *H. charlesbogerti* from its most closely related species, and a historical estimation of effective population size. We also provide information on the constraints of proteins in the Guatemalan Beaded Lizard and the presence of venom genes involved in defensive behaviors of this species. As additional sequences become available from highly endangered animals of related taxa, we hope that these novel results will provide avenues to research and conservation in this and other squamate species.

## Supplementary Material

jkac276_Supplementary_Data

## Data Availability

The assembled Guatemalan Beaded Lizard genome is available from https://doi.org/10.25387/g3.20092538. All PacBio raw long-read sequences used to generate the assembly are available from the National Center for Biotechnology Information (NCBI) Sequence Read Archive (SRA) database under the accession PRJNA834834 in the NCBI BioProject database (https://www.ncbi.nlm.nih.gov/bioproject/). Annotation of repeat regions, protein-coding regions, and functional annotation of predicted proteins are also available from the following figshare repository: https://doi.org/10.25387/g3.20092538. Supplemental material is available at G3 online.
